# Supplementary material for: Two Novel Mouse Models of Duchenne Muscular Dystrophy with Similar Dmd Exon 51 Frameshift Mutations and Varied Phenotype Severity
Source: Int J Mol Sci. 2024 Dec 27;26(1):158. doi: 10.3390/ijms26010158 (PMC11719507; doi:10.3390/ijms26010158)
Supplement: Supplementary file 1 [file ijms-26-00158-s001.zip › dmd Supplementary Materials.docx]

Supplementary Materials

Regaud’s iron hematoxylin staining for early myocardial damage

“MedX” (Moscow, Russia) Regaud’s iron hematoxylin staining kit is used in the study.

Its composition is as follows:

1. Regaud’s “MedX” mordant - homogenous transparent yellow-brown odorless solution. Is used as Weigert’s hematoxylin A oxidant. Not to be used without Weigert’s hematoxylin A.
2. “MedX” Weigert’s hematoxylin A - homogenous opaque brown solution with isopropanol smell. Used as a primary dye to stain nuclei and other cellular structures.
3. “MedX” Regaud’s differentiation solution - homogeneous transparent light-brown odorless solution. Used to remove the excessive Weigert’s hematoxylin A.

The mechanism of interaction between hematoxylin and trivalent iron ions is not well understood. It is known that iron ions act as oxidants and as a mordant and thus the solution containing the mordant and the dye will be active only for a limited time. The correct selection of dye-to-mordant ratio is essential. Iron hematoxylin staining is more stable against weak acidic solutions, and thus is considered acid-resistant. Mordant makes the tissue more receptive to alcohol hematoxylin solution, and the differentiation solution removes the dye from the tissue due to its weak binding with the tissues.

Cell nuclei, calcium deposits, fragments of muscle fibers with metabolic disruptions and some other structures are stained blue-black with hematoxylin within 3 to 10 minutes.

1. Deparaffinize and rehydrate the sample with distilled water
2. Place the slides into the humidity chamber. Apply the mordant and keep at +37C for 1 hour
3. Wash in three changes of distilled water, 2 minutes each
4. Apply Weigert’s hematoxylin A for 10 minutes at room temperature
5. Wash in distilled water for 2 minutes
6. Apply differentiation solution for 30-120 seconds, until the gray color develops. Monitor the color change under the microscope.
7. Wash in distilled water for 2 minutes
8. Dehydrate, clear in xylene, mount


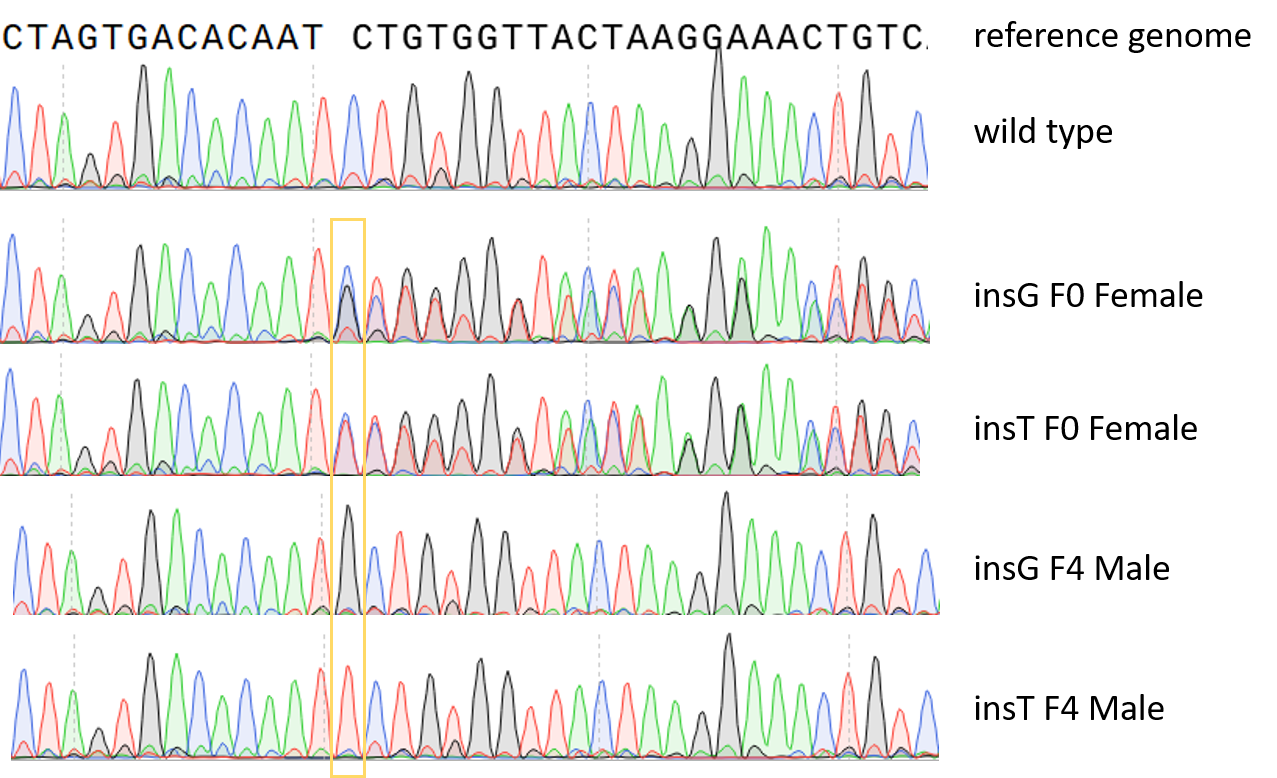


**Figure S1.** Sequence traces of *Dmd* mutation region in wild-type sample, and F0 and F4 generation mice samples. Yellow box corresponds to nucleotide position, at which insertions T or G occurred in insT and insG lines, respectively.


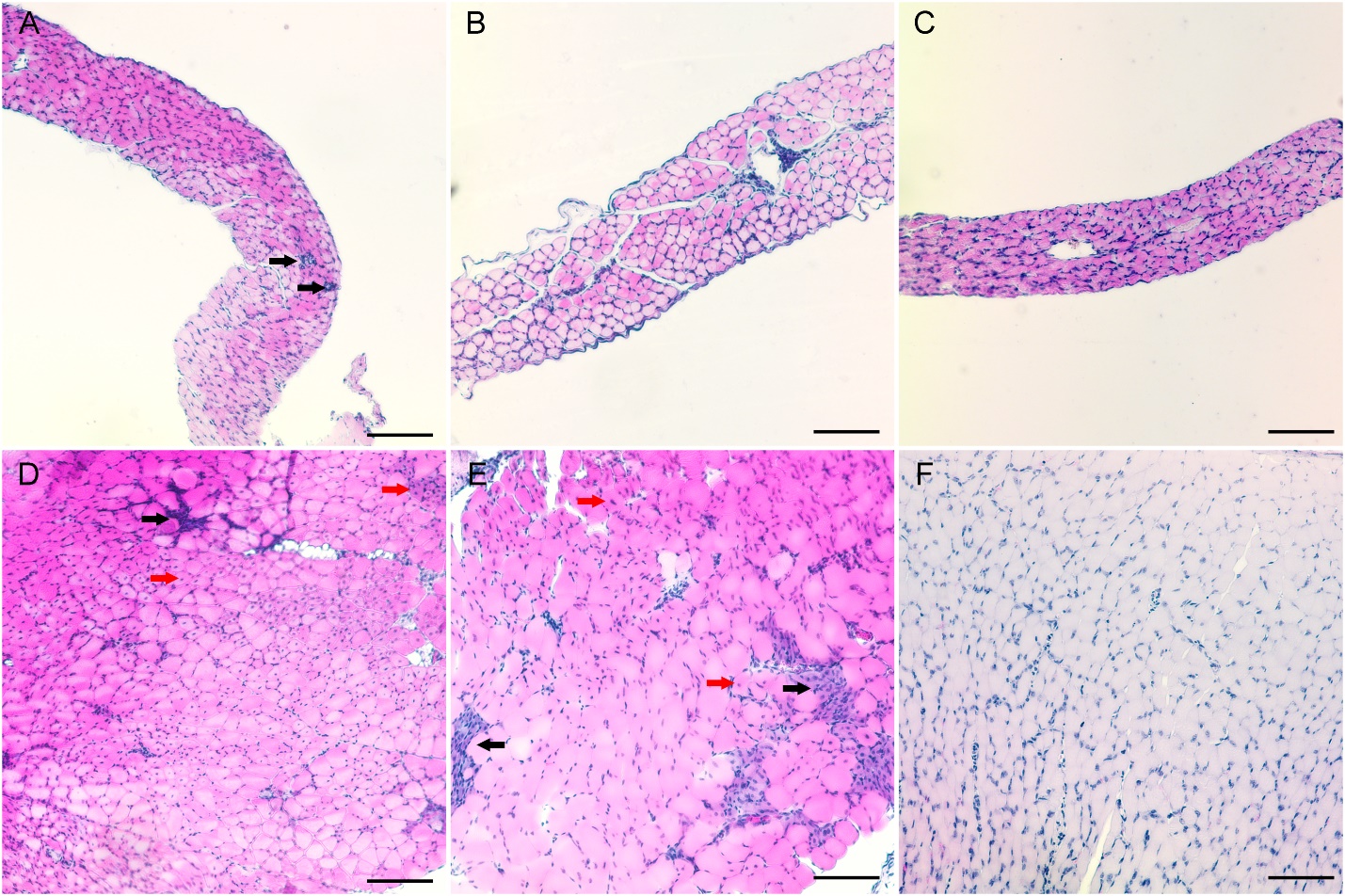


**Figure S2.** Cross sections of diaphragms (**A**–**C**) and skeletal (gastrocnemius) muscles (**D**–**F**), H&E staining. (A, D) — insG 1 week old mouse, (B, E) — insT 3 weeks old mouse, (C, F) — wild-type mouse. Black arrows — fibrosis and necrotic muscle fibers, red arrows — centrally nucleated fibers. Scale bar: 100 μm.

**
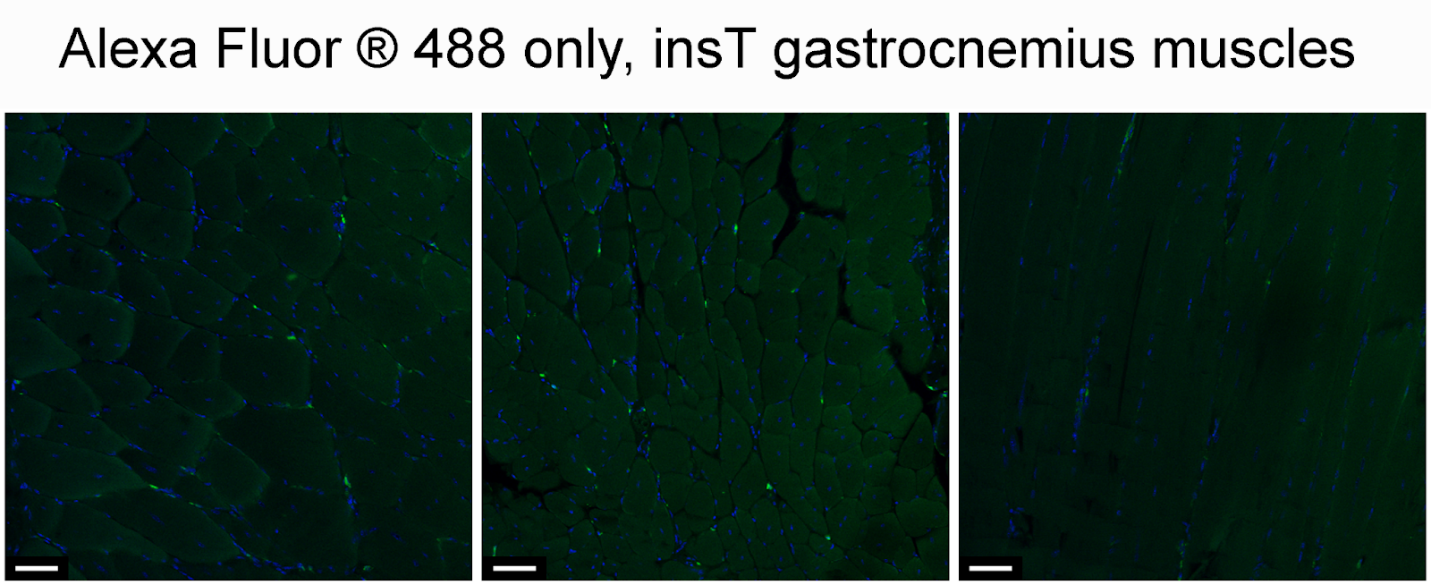
**

**Figure S3.** Immunofluorescence staining on FFPE tissues, insT gastrocnemius muscles. Alexa Fluor ® 488 only. Nuclear staining: Hoechst 33342 . Scale bar: 50 μm
